# Supplementary material for: PHLDA1 Does Not Contribute Directly to Heat Shock-Induced Apoptosis of Spermatocytes
Source: Int J Mol Sci. 2019 Dec 30;21(1):267. doi: 10.3390/ijms21010267 (PMC6982182; doi:10.3390/ijms21010267)
Supplement: Supplementary file 1 [file ijms-21-00267-s001.pdf]

PHLDA1 Does Not Contribute Directly to Heat Shock-Induced Apoptosis of Spermatocytes

Patryk Janus †, Katarzyna Mrowiec †, Natalia Vydra †, Piotr Widłak, Agnieszka Toma-Jonik, Joanna Korfanty, Ryszard Smolarczyk and Wiesława Widłak *

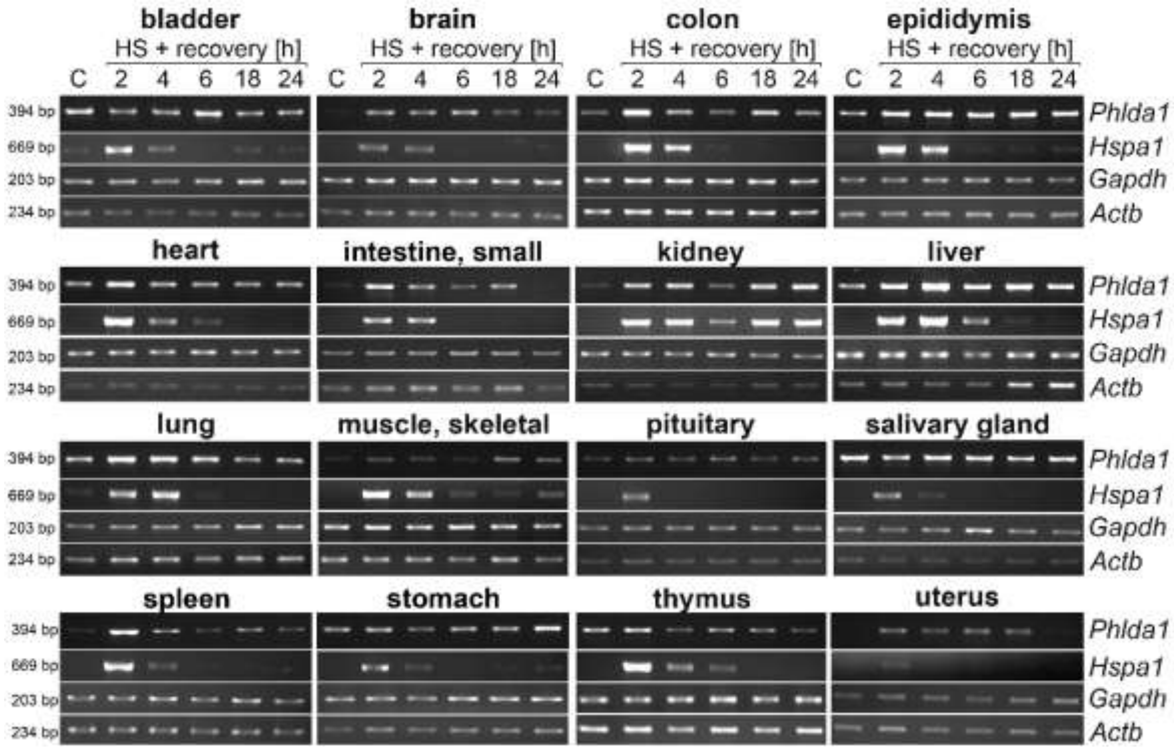

Figure 1. *Phlda1* transcript levels analyzed by RT-PCR in mouse organs after heat shock performed *in vivo* and indicated recovery time. C, control, physiological temperature; HS, heat shock. *Hspa1* was used as transcript level control for the heat shock response, *Gapdh*, *Actb* – as transcript level controls for loading.

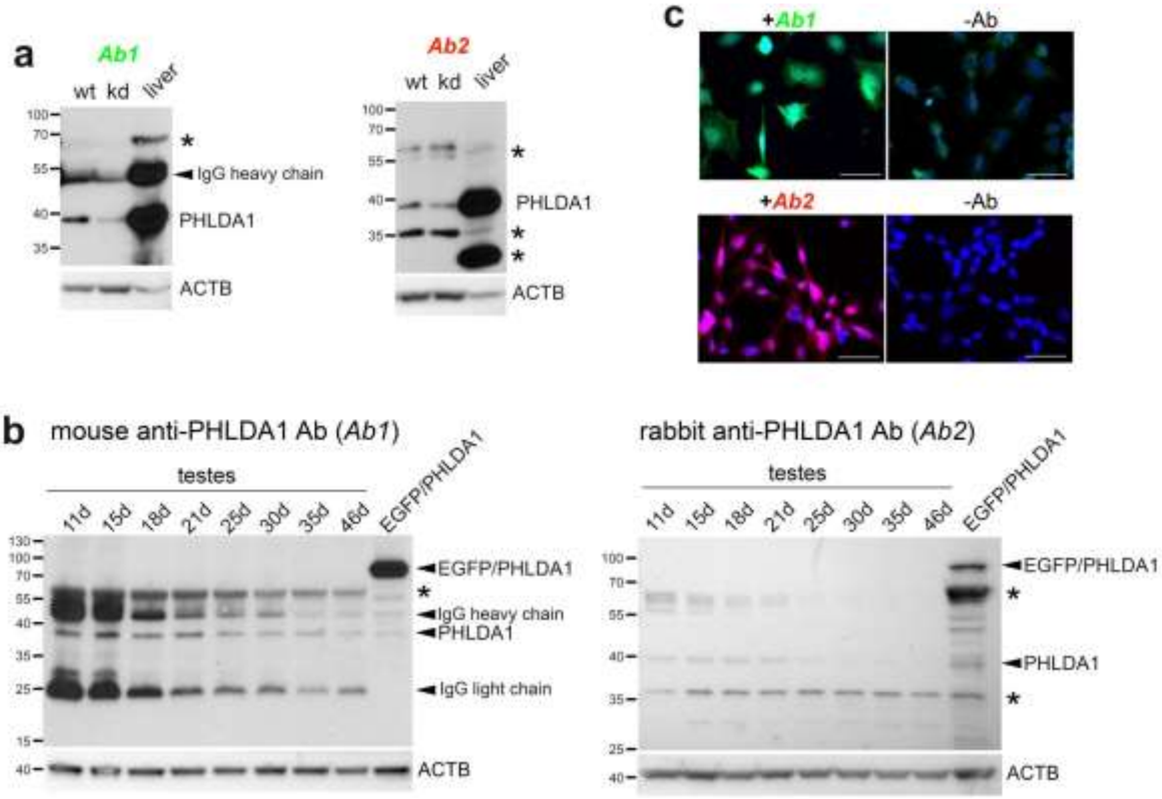

Figure 2. Specificity of anti-PHLDA1 antibodies: mouse monoclonal (Santa Cruz, sc-23866; *Ab1*) and rabbit polyclonal (Novus #NBP1-84969, *Ab2*). (**a**) PHLDA1 expression was reduced by sgRNA/Cas9 (kd, PHLDA1 knockdown; wt, wild type) in mouse HECa10 cells and analyzed by western blot. (**b**) Expression of PHLDA1 analyzed by western blot in the mouse testes during postnatal development and NIH3T3 cells transiently transfected with a vector coding for EGFP/PHLDA1 fusion protein. ACTB was used as a loading control. Both antibodies recognized the reduced level of PHLDA1 protein after PHLDA1 sgRNA knockdown and recognized EGFP/PHLDA1 fusion protein after its overexpression. Using *Ab1*, a higher background is likely on mouse tissues (IgG heavy and light chains were stained in western blot). In addition, a few other protein bands were detected by both antibodies (marked by asterisks); however, these bands would not be observed if cells with a high level of PHLDA1 expression (e.g., mouse hepatocytes) were analyzed using a shorter time of western blot exposure (not shown). (**c**) Detection of the PHLDA1 protein using *Ab1* (green) and *Ab2* (red) by immunofluorescence in HECa10 cells cultured *in vitro* (cells were fixed with 10% buffered formalin or 4% PFA for 10-15 minutes). Negative controls were performed for specific labeling by omitting the primary antibody (-Ab). DNA was stained with DAPI (blue). Scale bar – 50 μm. Both antibodies recognized endogenous PHLDA1 protein in cytoplasm and nuclei, which resembled the cellular distribution of EGFP/PHLDA1 fusion protein (Figure 3 in the manuscript), however using the mouse *Ab1*, a background is possible on mouse cells (even when Mouse on Mouse Kit is used for detection).

Table 1. Characteristics of primers used in RT-PCR analyses.

Gene Symbol	NCBI Reference Sequence	Primers Sequences	Product Lenght [bp]
Dazl	NM_010021.5	F: tgaagttgatccaggagctg<br>R: ccccctgagatgagttagca	261
Hspa1a<br>*Hspa1b*	NM_010479.2<br>NM_010478.2	F: ccatccagagacaagcgaag<br>R: cgtttagaccggcgatcac	699
Hspa2	NM_008301.4	F: agggcccaccatcgaggaag<br>R: gtacatggagatttgcttga	344
HSF1	NM_005526.2	F: ccagcaacagaaagtcgtca<br>R: gagctcattcttgtccaggc	325 in mutant *HSF1*
Pgk2	NM_031190.2	F: ggccctcagcaacatgtaat<br>R: aggactgtgggaaatcctga	228
Phlda1	NM_009344.3	F: caacagctccactcctaccc<br>R: gcttcctgcaactgtgatga	394
		F: ccgggccactcaaggttttg<br>R: actacttgatcaggcgcggg	112
Gapdh	NM_008084.3	F: tggtgaagcaggcatctgagg<br>R: catgaggtccaccaccctgt	203
Actb	NM_007393	F: ggacttcgagcaagagatgg<br>R: agcactgtgttggcgtacag	234
Hnrnpk	NM_001301341.1	F: tgggttcagtgctgatgaaa<br>R: aataggtccgccaagatcac	151
